# Decoding multifarious role of cow dung bacteria in mobilization of zinc fractions along with growth promotion of *C*. *annuum* L.

**DOI:** 10.1038/s41598-019-50788-8

**Published:** 2019-10-02

**Authors:** Kalpana Bhatt, Dinesh Kumar Maheshwari

**Affiliations:** Department of Botany and Microbiology, Gurukul Kangri University, Haridwar, 249404 Uttarakhand India

**Keywords:** Applied microbiology, Soil microbiology

## Abstract

Zinc is one of the micronutrients, required by all types of crops. About 10–100ppm of zinc is present in soil which is generally immobile. The cow dung sustains all life and being practice since aeons. Exploitation of cow dung bacteria can mobilize nutrients besides contributing in sustainable agriculture. Therefore, to examine mobilization of Zn, cow dung is used as a source of bacteria. The objectives of the present study were to isolate an array of bacteria from cow dung and to characterize them for their Zn (ZnO and ZnCO_3_) mobilization ability in addition to establish the optimum conditions for dissolution of zinc. A total of seventy bacterial isolates have been screened for Zn mobilization. Out of which most potent (CDK15 and CDK25) were selected to study the effect of various parameters viz. pH, temperature and concentration of Zn. These parameters were assessed qualitatively in diverse growth medium and quantitatively using Atomic absorption spectroscopy. Optimum pH and temperature for mobilization was recorded at pH 5 (ZnO) and 37 °C (ZnCO_3_) by CDK25, whereas, optimum zinc concentration for mobilization was recorded at 0.05% (ZnO) by CDK15. Maximum amount of Zn solubilized was recorded by CDK25 in ZnO (20ppm). Considering the abilities of most potent bacterial isolates with reference to P-mobilization and growth promoting traits, pot culture assay of *C*. *annuum* L. was carried out. The findings of which conclude that, bacterium CDK25 (*Bacillus megaterium*) could be exploited for factors viz. nutrient management of Zn, growth promoting agent, and Zn augmentation in soil.

## Introduction

Among all micronutrients, Zn is indispensible, for various physiological and biochemical functions in all types of crops. Improper supply of which can directly effects the growth and yield of plant. Generally, soils are rich in zinc, but are mainly associated with hydrous Fe^3+^ and Al^3+^ oxides. So, release of which in fixed and immobilized, a major aspect adversely affecting soil quality. Mobilization of Zn by bacteria is carried out which transfers unavailable fraction to available one. As per Vedic scriptures, Gomeya/Cow dung are not a waste product but do purify all the waste present in the nature^1^. Cow dung microflora includes about 60 bacterial species dominating by *Bacillus* sp., *Corynebacterium* sp., *Lactobacillus* sp., few fungal sp., (*Aspergillus* and *Trichoderma*), about100 species of protozoa and 2 yeasts. Studies revealed that it contains diverse group of bacteria viz., *Acinetobacter*, *Serratia* and *Alcaligenes* spp.^2–4^ as well as plant growth promoting bacteria. Plant growth promoting bacteria consist of mechanisms contributing in plant growth and yield; one of them is direct mechanism. A mechanism influencing plant growth by Zn-solubilization, P-solubilization, phytohormones production (IAA), siderophore and HCN production^5^. Plant growth promoting bacteria enhances the plant vegetative and reproductive growth parameters by colonizing plant roots. Further establishing symbiotic association with plants, leading to enrichment of soil vital nutrients viz., nitrogen (N), by fixing it from the atmosphere, phosphorous (P), potassium (K) by solubilizing it from the soil^6–8^, along with enhancing the plant growth. Besides providing major plant nutrients, plant growth promoting bacteria also aid in providing soluble zinc (ZnO, ZnCO_3_, and Zn_3_ (PO_4_)_2_) from total soil zinc^9,10^. The soluble form of zinc is further utilized to increase the availability to plants^11^. The effectiveness of these bacteria is via their associations with plant roots, which involves processes viz., solubilization, mobilization, mineralization, biofortification of the zinc pool from soil to plant cells. The zinc deficiency is a major problem leading improper plant growth and degradation of soil quality. The cow dung inhabiting bacteria mobilize insoluble form of Zn in soil, making them easily available for plants.

*C*. *annuum* L. is the crop which is consumed in both forms, fresh and dry, having high nutritional, commercial and medicinal value. In this light, the present study on growth enhancement of *C*. *annuum* L. becomes very crucial. Hence, our endeavor through this study is to isolate an array of bacteria from cow dung followed by identification and preliminary screening with reference to Zn mobilization. In addition to establish its optimum dissolution mechanism, whether increased, decreased or neutral pH, temperature and concentration of Zn alters mobilization potential, followed by establishing cow-dung inhabiting wild bacteria interaction in relation to enhancement of growth and productivity of *C*. *annuum* L.

## Material and Methods

### Dung collection and analysis

A total of seven cow dung samples, i.e. fresh (morning time) and dry (randomly) were collected from seven different locations of Haridwar and Dehradun (Uttarakhand, India). Dung samples were collected from different age categories viz., young ones (3–7 months old), lactating cows (11–16 months old) and non-lactating cow, generally after parturition in a sterile container and aseptically brought to the Microbiology Laboratory of the University for further analysis^12^. Sample collected from all the cows was routinely fed with locally available green fodder along with the silage and crop residues. Physio-chemical analysis of dung was carried out in which organic carbon was estimated by digestion, phosphorous content by Olsen method, potassium by flame photometer, zinc by Atomic absorption spectroscopy (Table 1).

**Table 1:** Physio-chemical characteristics of cow dung.

S.N.	Parameters	Value
1	pH	7.1 ± 0.033
2	Electric conductivity (ds/m)	0.97 ± 0.005
3	Organic carbon (%)	26.27 ± 0.005
4	Nitrogen (%)	0.924 ± 0.007
5	Phosphorus (%)	0.274 ± 0.003
6	Potasium (%)	1.025 ± 0.003
7	Zinc (%)	0.123 ± 0.001

All the values are arithmetic mean of three replicative observations with ±SE.

### Analysis of soil samples

Soil samples were randomly collected from different locations and properly mixed. Thereafter their physio-chemical characteristics were analyzed, where estimation of organic carbon, phosphorous, potassium, zinc and magnesium content was carried out. Analysis of both dung and soil was carried out at Indian Institute of Soil and Water Research Conservation, Dehradun, India (Table 2).

**Table 2:** Analysis of soil samples.

S.N.	Parameters	S1	S2
1	pH	7.34 ± 0.026	7.26 ± 0.030
2	Electric conductivity (ds/m)	0.16 ± 0.020	0.17 ± 0.015
3	Organic carbon (%)	1.12 ± 0.015	1.14 ± 0.015
4	Phosphorus (mg/kg)	53.76 ± 1.708	71.68 ± 0.995
5	Potassium (mg/kg)	172.6 ± 1.069	548.4 ± 1.069
6	Zinc (ppm)	4.10 ± 0.005	4.91 ± 0.020
7	Magnesium (ppm)	6.90 ± 0.036	17.34 ± 0.073

All the values are arithmetic mean of three replicative observations with ±SE (where, S1: Soil analysis at 0 days after sowing; S2: Soil analysis at 120 days after sowing).

### Isolation and identification of bacterial isolates

Isolation of bacteria from dung was carried out by serial dilution method^13^. Physiological, morphological and biochemical characterization of all the bacterial isolates were done on different media (NAM, BAM, MHA and LB medium) by following the criteria described in Bergey’s Manual of Bacteriology^14^.

### Zinc solubilization

Qualitative screening of selected isolates for Zn mobilization was carried out in Bunt and Rovira medium agar plates having 0.1% insoluble zinc sources (ZnO and ZnCO_3_). Isolates were spot inoculated into the media and incubated at 30 ± 1 °C for 24–48 hours. Zinc solubilization was observed by measuring clear zones around the colonies. Quantification of Zn mobilization in liquid medium was done by using same chemicals as in qualitative assay. The availability of free Zn was estimated by using Atomic absorption spectroscopy^15^.

### Optimization of growth parameters effecting zinc solubilization

Optimization of growth parameters was carried out to study the maximum efficiency and amount of zinc solubilized.

#### Effect of different concentration

Effect of different concentration of both Zn sources was added in the Bunt and Rovira agar medium which wa**s** 0.5%, 0.1%, 0.15%, 0.2% and 0.25%. Inoculation was carried out by using pure colony of selected bacteria. It was inoculated to medium and allowed to grow at 37 ± 1 °C for a week. Solubilization was monitored every 24 hours of interval.

#### Effect of temperature

Inoculation was carried out in Bunt and Rovira agar medium amended with Zn sources using pure colony of selected bacteria and maintained at 37 °C, 30 °C and 27 °C for a week.

#### Effect of pH

Effect of different pH on zinc solubilization ability by bacterial isolates was observed. Bacterial isolates were inoculated in the Bunt and Rovira agar medium amended with Zn sources of which pH was set as: pH 5, pH 6, pH 7, pH 8 and pH 9 and were incubated for about a week respectively.

### Evaluation of nutrient solubilization and growth promoting traits (PGP)

Cow dung bacterial isolates showing maximum Zn solubilization were assessed for PGP traits like P-solubilization, Indole acetic acid (IAA) and HCN production.

#### Phosphate solubilization

Qualitative and quantitative P-solubilization by bacterial isolates was estimated by spot inoculating them on Pikovskaya’s agar medium at 28 ± 1 °C, 48–72 hours. Further phosphate solubilization index was evaluated as described by Dubey and Maheshwari^13^.

#### Indole-3-acetic acid (IAA) production

The bacterial isolates were grown on LB broth, incubated at 28 ± 1 °C, 24–48 hours and were centrifuged at 10,000 rpm for 15 min. Salkowaski reagent was added in supernatant (2 ml); appearance of pink colour confirmed the production of IAA. Quantification assay of the IAA production was further also carried out^16^.

#### HCN production

HCN production was determined by streaking the bacterial isolates on LB agar plates supplemented with 4.4 g/l glycine. Filter paper soaked in 0.5% picric acid in 1% Na_2_CO_3_ was added in the upper lids of plates along with control and plates were sealed with parafilm, incubated at 28 ± 1 °C; development of colour from yellow to light brown, moderate brown or strong brown was examined for HCN production^17^.

### Molecular characterization and phylogenetic analysis

Most promising bacterium was subjected to molecular identification by 16S rRNA gene sequencing. Genomic DNA was isolated according to Sambrook and Russel^18^. Amplification of 16S rRNA gene of CDK25 was carried out by PCR using primers 27F 5′ (AGA GTT TGA TCM TGG CTC AG) 3′ and 1492R 5′ (TAC GGY TAC CTT GTT ACG ACT T) 3′. The sequence homology was studied by BLAST n search program of GenBank database (NCBI) and was aligned by Clustal W. Phylogenetic tree was constructed using the neighbour-joining method by MEGA6 software.

### Pot culture assay

Pot culture assay was carried out to study the effects by proficient isolates (CDK15 and CDK25) on vegetative plant growth parameters of *C*. *annuum* L. Healthy seeds of same shape and size were surface sterilized with 95% ethanol (30 sec) followed by 4% NaOCl treatment (2–3 min). Further seeds were washed with sterile distilled water (SDW) and were allowed to dry overnight. The seed bacterization was done according to Weller and Cook^19^. Seeds were bacterized using 1% carboxy methyl cellulose (CMC) slurry for coating the seeds surface and the potential isolates (CDK15 and CDK25) were mixed and sown in pots (12″ diameter) containing pre-sterilized soil, with following sets of treatments: T1- seeds without bacterial inoculant coating; as control, T2- seeds treated with CDK15, T3- seeds treated with CDK25, T4- seeds treated with Consortium (CDK15 + CDK25). The growth parameters like plant height, number of branches, root length, fresh and dry root weight, number of fruits and fruits yield data were recorded at 30, 60, 90 and 120 days after sowing (DAS).

### Statistical analysis

Effect of pH and temperature data was subjected to principal component analysis (PCA) to determine the statistical correlation between the different treatments of ZnO and ZnCO_**3**_ by using XLSTAT software. Probit analysis was determined to study the effect of concentration on mobilization efficiency of ZnO and ZnCO_**3**_ by BioStat v5 software. The cow dung and soil analysis data so obtained are expressed as mean of three replicates with ± standard error of mean. Likewise, pot culture assay were conducted in triplicate and mean data ± SE (standard error) were reported. The data obtained were subjected to one-way analysis of variance (ANOVA). Significance between treatments was determined by Duncan’s multiple range tests at *p < *0.05, by using statistical package for the social sciences (SPSS) version 20.0 software.

## Results

A total of 70 bacteria were isolated from “Desi” cow dung, designated as CDK. On the basis of physio-morphological and biochemical characterization, 32 were found as Gram negative cocci, 18 as Gram negative rods, 11 as Gram positive rods and 09 as Gram positive cocci. Proficient isolates were selected for further study (Table 3).

**Table 3:** Physio-morphological and biochemical characterization of potential zinc solubilizing bacterial isolates.

S.N.	Assay		CDK15	CDK25
1	Colony morphology		Cream coloured, moderate	Cream coloured, large
2	Gram’s reaction		+ve	+ve
3	Endospores		+ve	+ve
4	Shape		rods	rods
5	Catalase test		+ve	+ve
6	Indole		−ve	−ve
7	Methyl red		−ve	+ve
8	Vogues Proskauer		−ve	−ve
9	Citrate utilization		−ve	−ve
10	Nitrate reduction		+ve	+ve
11	Malonate		+ve	+ve
12	ONPG		+ve	+ve
13	Phenylalanine deaminase		+ve	+ve
14	H_2_S production		−ve	−ve
15	Utilization of	Lysine	+ve	+ve
		Ornithine	+ve	+ve
16	Hydrolysis of	D-Glucose	+ve	+ve
		Starch	+ve	+ve
		Gelatin	+ve	+ve
17	Growth in	NAM	+	+
		LB	++	++
		BAM	++	+
		MHA	++	+++

All the assays were performed in triplicates (+ve/+positive reaction, −ve negative reaction, +++ maximum, ++ moderate, + minimum).

### Zinc solubilization

**Figure 1:**
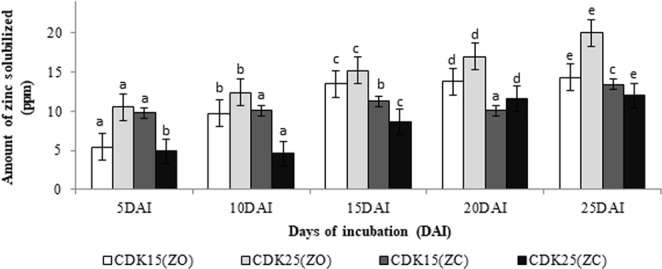
Amount of zinc solubilized (Where, ZO: Zinc oxide; ZC: Zinc carbonate). Values are mean of three replicates, vertical bars in the column representing standard error of mean. Values with different superscript (**a**–**e**) on a column are significantly different as determined by Duncan’s multiple range tests (p < 0.05).

From all the isolates, only 8 were able to produce zone of clearance in zinc oxide and zinc carbonate. Maximum solubilization index and solubilizing efficiency occurred due to CDK25, along with 20.0 ppm solubility in ZnO and 14.01ppm in ZnCO_**3**_ by CDK15 (Fig. 1).

### Optimization of growth parameters effecting zinc solubilization

#### Effect of different concentration

**Figure 2:**
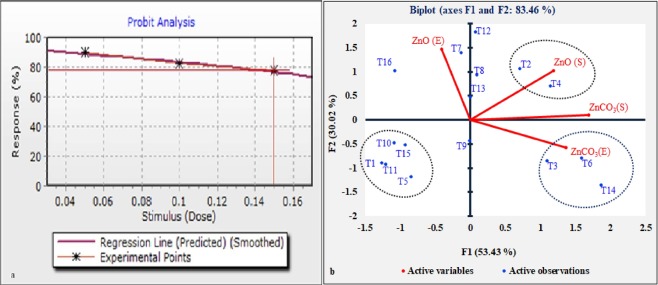
(**a**) Effect of different Concentration on Zinc solubilization. (**b**) Principal Component Analysis showing correlation between effect of different pH and temperature on Zinc solubilization. [Where, ZnO(E); ZnO(S):Solubilizing Efficiency and Amount solubilized in ZnO respectively, ZnCO3(E); ZnCO3 (S):Solubilizing Efficiency and Amount solubilized in ZnCO3 respectively. T1: CDK15 (27 °C), T2: CDK15 (30 °C), T3: CDK15 (37 °C), T4: CDK25 (27 °C), T5: CDK25 (30 °C), T6: CDK25 (37 °C), T7: CDK15 (pH5), T8: CDK15 (pH6), T9: CDK15 (pH7), T10: CDK15 (pH8), T11: CDK15 (pH9), T12: CDK25 (pH5), T13: CDK25 (pH6), T14: CDK25 (pH7), T15: CDK25 (pH8) and T16: CDK25 (pH9)].

In the present study 0.5%, was found to be optimum concentration for obtaining maximum efficiency by CDK15 (143.75%) in ZnO followed by 0.1% (CDK25-127.77%) in ZnCO_**3**,_ whereas 0.15% was found to be least concentration. No zone of clearance was observed at 0.2% and 0.25% (Fig. 2a and Table 4). The data of concentration were statistically evaluated by probit analysis, which reveals 0.15% to be threshold concentration for solubilization. Hence, the concentration results of zinc shows the tolerance level of the bacterial isolates.

**Table 4:** Probit Analysis - Least squares [Normal Distribution].

Dose(Stimulus)	Actual Percent (%)	N	Probit (Y)	Weight (Z)
0.05	0.896666666666667	300	6.26294937910481	2.71115186268558
0.1	0.82	300	5.9152629999485	3.669474000103
0.15	0.773333333333333	300	5.74962838446414	4.00074323107173

#### Effect of temperature

The effect of different temperature (27 °C, 30 °C, and 37 °C) was carried out in order to observe the optimum temperature at which best solubilization takes place at, 7days. 37 °C, was found to be optimum temperature for obtaining maximum efficiency by CDK25 (182.35%) in ZnCO_3_. Whereas, 30 °C for solubilized amount by CDK15 (19.3 ppm) in ZnO (Fig. 2b.)

#### Effect of pH

In the present study the selected isolates were able to grow at different pH (5, 6, 7, 8 and 9). CDK25 solubilized maximum zinc in pH 7(17.2ppm) with maximum efficiency (205.26%). The pH 9 was found to be least for both isolates. Principle Component Analysis (PCA) of pH and temperature revealed statistical correlation between different treatments and variables. The PCA of different variables is explained with component 1 (F1:53.43%) and component 2 (F2:30.02%) (Fig. 2b).

### Evaluation of nutrient solubilization and growth promoting traits (PGP)

All the eight zinc solubilizing bacterial isolates showed P-solubilization ranging with (2.45–3.46 cm) of solubilization index within a week, at 37 ± 1 °C. Further quantification assay showed the varying levels of phosphate solubilization in liquid medium. Five isolates showed IAA production which was confirmed by quantification assay. Maximum IAA production was observed by CDK25 (13.8 μg/ml), followed by CKD15 (11.6 μg/ml). Out of all isolates, four produced HCN (Table 5).

**Table 5:** Nutrient solubilization and growth promotion traits of potential zinc solubilizing bacteria.

Isolates	Nutrient solubilization trait		Growth promotion trait	HCN production
	Phosphate		IAA (μg/ml)	
	Solubilization index(cm)	Solubilized (mg/ml)		
CDK15	3.46	264.04	11.6	+
CDK25	3.30	281.59	13.8	++

Abbreviation: ++ maximum, + moderate. All the assays were performed in triplicates.

### Molecular characterization and phylogenetic analysis

**Figure 3:**
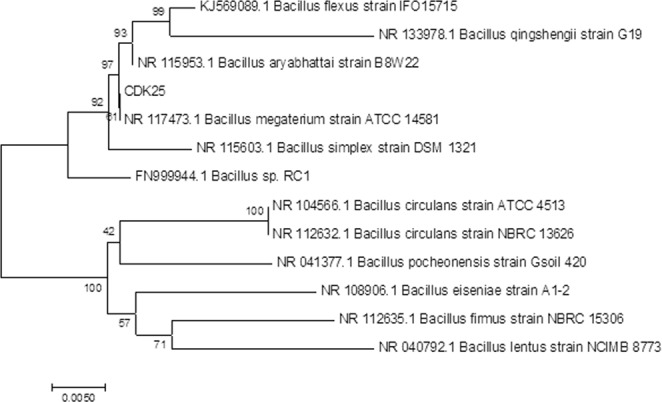
Evolutionary relationships of taxa.

The most potential isolate CDK25 was identified by 16S rRNA sequencing. Nucleotide sequences have been submitted to the GenBank nucleotide sequence database under the accession number (MG774438). A nBLAST search for the 1446 bp 16S rRNA nucleotide sequence of isolate was phylogenetically 97% similar to *B*.*megaterium* ATCC 14481 (Accession number NR113670) (Fig. 3).

### Pot culture assay

**Figure 4:**
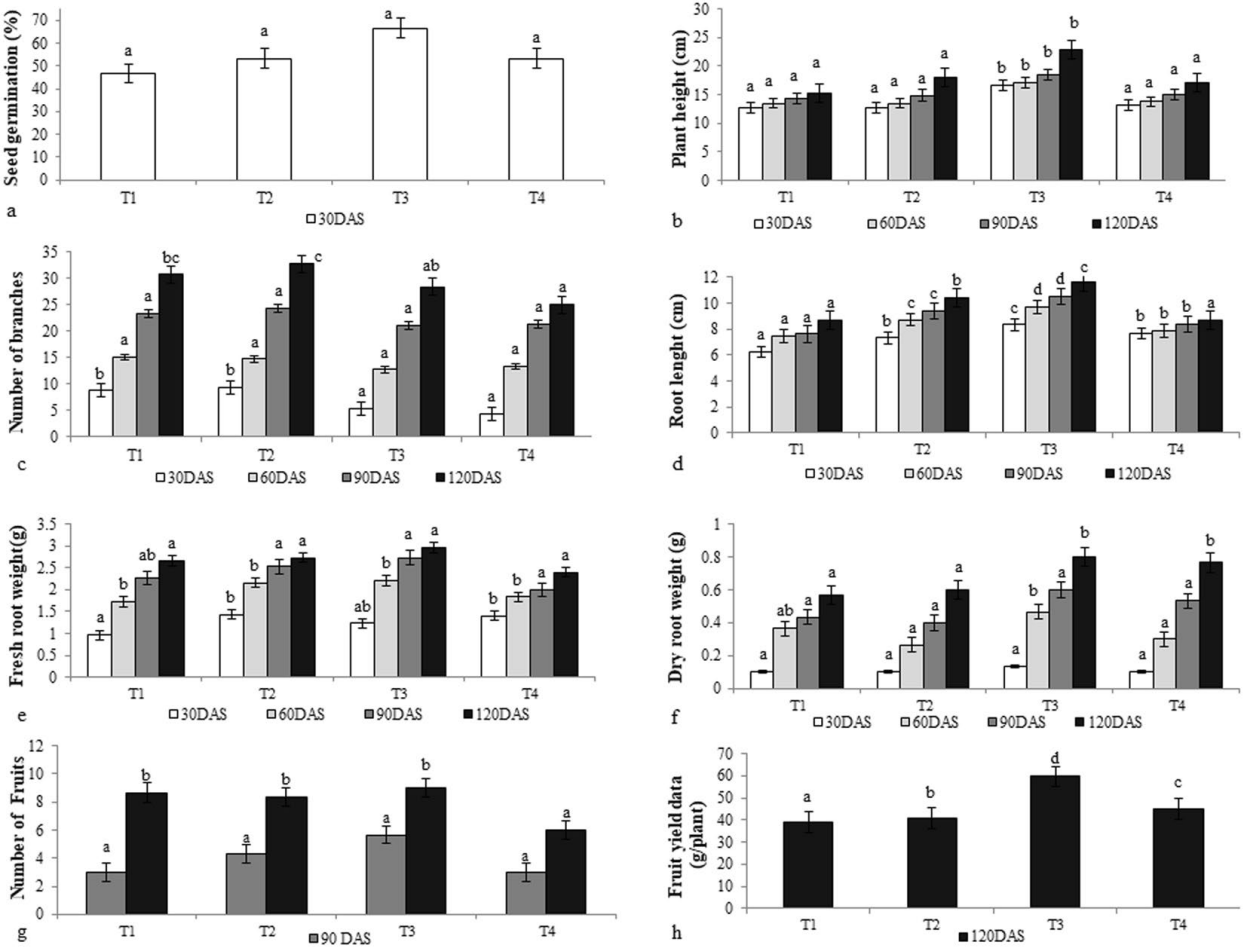
Effect of bacterial isolates on (**a**) Seed germination rate (**b**) Plant height (**c**) Number of branches (**d**) Root length (**e**) Fresh root weight (**f**) Dry root weight (**g**) Number of Fruits (after 120 DAS) (**h**) Fruit yield data of *C. annuum* L. (after 120 DAS); Where, T1: Untreated control, T2: seeds + CDK15, T3: seeds + CDK25, T4: seeds + Consortium (CDK15 + CDK25), DAS: days after sowing. Values are mean of three replicates, vertical bars in the column representing standard error of mean. Values with different superscript (a-d) on a column are significantly different as determined by Duncan’s multiple range tests (p < 0.05).

Among all the treatments, maximum plant height was recorded by treatment T3-CDK25 with a value of 22.86 cm followed by T2-CDK15 with a value of 18 cm after 120DAS. Maximum number of branches/plant was observed by T2-CDK15 (32.66) followed by T1-Control (30.66) and T3-CDK25 (28.33) after 120DAS, maximum root length, fresh and dry root weight was observed by T3- CDK25, whereas, treatment T3 also showed the maximum number of fruits and yield followed by T1-control and T4-(CDK15 + CDK25) respectively after 120DAS (Fig. 4).

## Discussion

Since ages, Indian farmers are practicing cow dung as organic agricultural fertilizer. Addition of cow dung is known to enrich the soil nutrient status and enhancement of plant growth parameters. But, its direct application is unsuitable for soil nutrient profile. Besides, consisting minerals, fibers and crude protein, cow dung also consist of beneficial microflora, predominantly bacilli, lactobacilli, cocci and some identified and unidentified fungus and yeast as reported by Muhammad and Amusa^20^. However, exploitation of microflora from cow dung for plant growth enhancement and phosphate solubilization^21^, cellulase producing bacteria^22,23^, enzymatic activities^24^, methanogenic bacteria^25^, antibiotic resistant strains^26^, antibiotic susceptibility^27^ and ammonia producing bacteria are well reported^28^.

Zinc is an important micronutrient, having multiple roles in plants life cycle viz. growth, maturity, vigor and yield. Mobilization of zinc varies in plants, depending on its availability in the soil eco-system. When zinc is inadequate, its translocation is delayed from older to younger leaves, which leads to zinc deficient plant. So, the present study focuses on mobilization of zinc with respect to plant. On the contrary, availability of soluble zinc is low in soil, which degrades the soil fertility, besides triggering its uptake by plant. Therefore there is a need of identification of such noble bacterial isolates by which deficiency of zinc in soil and plant eco-system can be minimized. So, application of zinc mobilizing bacteria as a bioinoculant can transform the unavailable fractions of zinc into the available one. Since, studies on screening of zinc mobilizing bacteria have been widely studied^29–32^. But, there is lack of research related to isolation and characterization of the zinc mobilizing bacteria from cow dung with reference to nutrient solubilization and plant growth associated traits. Hence, the present investigation includes isolation, identification and screening of such bacteria from cow dung.

A total of 70 bacterial isolates were procured from the cow dung sample collected from different locations of Haridwar and Dehradun, Uttarakhand. On the basis of zinc mobilizing ability and nutrient solubilization (P) and growth promoting traits (IAA and HCN), most potent bacterial isolates designated as CDK15 and CDK25 were characterized based on physio- morphological and biochemical tests which confirmed them as *Bacillus* spp. These results get in support from the study of Swain and Ray^21^_,_ reported *Bacillus subtilis* from cow dung, capable to solubilizing phosphate. Our results of qualitative dissolution of zinc revealed that, zone of clearance was observed in both zinc sources by both the isolates. This was interesting to note that, increased dissolution was observed from quantitative assay, which state maximum solubility occurred after incubation up to 7days, by CDK25 in ZnO with zone of clearance (5.1 cm), solubilizing efficiency (364.28%) and amount of zinc solubilized (20ppm). Our results get in support by Saravanan *et al*.^15^, where zinc solubilization was observed by *Bacillus* spp. Both the bacterial isolates were further examined to solubilize nutrient (P) and revealed higher nutrient solubility (281.59 mg/ml) by CDK25 followed by CDK15 (264.04 mg/ml). The findings of P-solubilization by *Bacillus* spp. goes well with the findings of Dubey *et al*.^33^, who reported P-solubilization by *Bacillus subtilis* (BSK17), besides enhancing *Cicer aerietinum* yield. Maximum IAA production was observed by CDK25 (13.8 μg/ml) followed by CDK15 (11.6 μg/ml) was recorded. As a growth promoter hormone, maximum production of IAA by plant growth associated bacteria resultant to increase plant vegetative growth parameters. At particular concentration, IAA can alter young seedlings physio-morphologically^34^. Our reports agree with Radha and Rao^28^, who reported IAA production by cow dung bacteria. Similar study on IAA production was carried out by Pandey *et al*.^35^, where IAA production occurred by *B*. *subtilis* (9.5 μg/ml) and *B*. *pumilus* (7.9 μg/ml). Gonita-Mishra *et al*.^36^, also reported production of IAA with an range of 4.7–77.41 μg/ml.

Since, mobilization of zinc plays a vital role in nutrient cycling which releases zinc fractions into biogeochemical cycles; making it available for biological uptake. Since, up till now pH 7 was used in the zinc mobilization methodology. So, in the current study most potent Zinc mobilizing cow dung bacterial isolates, were further studied for optimization of parameters like pH (range 5–9), temperature (27 °C, 30 °C, and 37 °C) and different concentration (0.5%, 0.1%, 0.15%, 0.20% and 0.25%) of zinc. As, pH of the medium plays an important role in obtaining maximum solubilization. So, by findings, pH 5 appeared to be the optimum pH for maximum solubilization of ZnO by CDK25 (16.5ppm). Whereas, pH 7 appeared to be the optimum pH for maximum solubilization of ZnCO_3_ by CDK25 (17.2ppm) followed by pH 6 by CDK15 (12.4ppm). On the contrary, maximum efficiency was obtained at pH 7 (205.26%) in ZnCO_3_ followed by pH5 (147.05%) in ZnO by CDK25. However, both isolates solubilized zinc in pH 8 and 9, but with a minimum effect.

Each solubilizing bacterial isolates are sensitive to various environmental conditions, one of them is temperature. Similar to pH, effect of different temperature on solubilization was also evaluated. Where, 37 °C (182.35%) appeared to be the optimum temperature where maximum efficiency was obtained, followed by 30 °C in ZnCO_3_ by CDK25. 30 °C, appeared to be the most favorable temperature for amount of solubilization by CDK15 (19.5ppm) narrowly followed by 27 °C for CDK25 (19ppm) in ZnO. Whereas, 30 °C appeared to be least favorable for amount of solubilization by CDK25 (8.1ppm) in case of ZnCO_3_. Further, the effect of pH and temperature was studied by Principle Component Analysis (PCA). The analysis was carried out to evaluate the statistical correlation between different treatments and different variables (efficiency and amount of solubilization of ZnO and ZnCO_3_). The PCA of different variables is explained with component 1 (F1:53.43%) and component 2 (F2:30.02%). Analysis revealed that T-2 and T-4 were most effective to increase the solubilization amount of ZnO (S) as evidenced with positive correlation and appeared in the same group. However, T-3, T-6 and T-14 were also present in the same domain and positively correlated with the enhancement of efficiency of ZnCO_3_ (E). However, the negative correlation was observed between treatments T-1, T-5, T-10 and T-15, which shows fewer roles of these treatments on the solubilization of different source of zinc. PCA data depicts positive correlation between different treatments with different variables, which confirms the increment in mobilization of zinc sources by these bacterial isolates. Thus, the analysis data favored T-2 and T-4 was found to be the best treatment for amount solubilization of ZnO. Whereas, T-3, T-4 and T-14 was found be the best treatments for obtaining maximum mobilization efficiency of ZnCO_3_.

The data of effect of concentration on mobilization efficiency of ZnO and ZnCO_3_, obtained were statistically evaluated using probit analysis method. Mobilization efficiency decreases with increasing concentration of zinc sources (ZnO and ZnCO_3_). Probit analysis data depicts 0.05% concentration to be best for obtaining maximum mobilization, followed by 0.1% and 0.15%. However no growth and zone of clearance was observed in 0.2% and 0.25% zinc concentration. So, the results state that the concentration above 0.15% of Zn seems to be inhibitory for the bacterial growth showing no zone of solubilization. Which conclude 0.15% concentration to be the threshold concentration for obtaining solubilization.

Pot culture assay experiments are very much essential for analyzing the effects of bacterial isolates on various plant growth parameters. In the present study, effect of cow dung bacterial isolates (CDK15 and CDK25) mobilizing zinc fractions was examined in a pot culture assay on growth parameters of *C*. *annuum* L. experiment. Bacterial isolates CDK15 and CDK25 when used individually as well as in combination with each other improved growth of plants significantly over the control. Pot culture data suggested that vegetative growth parameters like plant height, number of fruits/plant and fruits yield were found to be superior in treatment T3-CDK25 over the T1-control and other treatments. This might be due to better nutrient mobilization by bacteria, making it avail in soil and thus their uptake by the leaves, roots and fruits of host plant resulting in increment of seedling growth. Earlier reports also supports such study, where plant growth promoting bacterial treatment has resulted in increased nutritive value, along with enhanced plant growth and yield^37,38^. Similar studies proving the correlation between bacterial application and increment in plant yield have been reported viz. Ramesh *et al*.^39^. Tariq and Ashraf^40^, also reported increment in grain yield by application of plant growth promoting zinc mobilizing bacteria. Iqbal *et al*.^41^ reported plant growth promotion of *Vigna radiata* by zinc and phosphate solubilizing bacteria. Recent studies also provide evidence that application of phosphate-zinc-solubilizing bacteria results in increment of chickpea grain yield^42^.

The identity of the selected potent isolate was confirmed by 16S rRNA gene sequencing and phylogenetic analysis which reveals CDK25 to be *Bacillus megaterium*. *B*.*megaterium* plant growth promoting abilities and formulation have also been studied by Trivedi and Pandey^43^. But, this is the first report on plant growth promotion and seed germination of *C*. *annuum* L. by *B*.*megaterium* with reference to zinc mobilization. Incorporation of *B*.*megaterium* can increase zinc transformation in soil by availing solubilized zinc to soil for growth and development of plants. Similar study on inoculation of zinc solubilizing bacteria i.e. *B*. *aryabhattai* and *Bacillus* sp. (PAN-TM1) to tomato seedlings helps in enhancing growth, yield and quality parameters of tomato^44^. To the best of our knowledge, this is the first report, suggesting role of cow dung bacteria as zinc mobilizer and plant growth promoter. This work may be a promising alternative to improve soil nutrient profile, besides contributing in development of sustainable agriculture.

## Conclusions

This is the first investigation which provides the detailed information of screening the cow dung bacterial isolates for dissolution of macro (P) and micronutrient (Zn) fractions, optimization of mobilizing factors and assessment of plant growth associated parameters of *C*. *annuum* L. Zinc solubilization by cow dung bacteria showing PGP traits is relatively a newer approach and most of the dung bacteria have not yet been tested for this activity. With the evident of results, it appears that zinc mobilization is a multifactor phenomenon which depends on several factors viz. pH, temperature and concentration of zinc. So, the prediction of optimum pH and temperature for micronutrient (Zn) dissolution besides establishing a threshold concentration for same was concluded in this report. Based on these dissolution parameters, ZnO was found to be solubilized readily by bacterial isolates. Evaluating pot culture assay results, it can be concluded that bacterium CDK25 enhances the growth of *C*. *annuum* L. Our findings suggested that bacterium isolated from cow dung can established potent role for preparation of bioinoculant or biofetilizer and lesson the dependency on costlier approaches for enhancement of plant growth.

## Data Availability

Nucleotide sequences have been submitted to the GenBank nucleotide sequence database under Accession codes MG774438.
